# Foreword Special Issue Cell Cycle and Regulation

**DOI:** 10.3390/genes11030254

**Published:** 2020-02-27

**Authors:** Ana María Zubiaga

**Affiliations:** Department of Genetics, Physical Anthropology and Animal Physiology, University of the Basque Country UPV/EHU, 48080 Bilbao, Spain; ana.zubiaga@ehu.eus

The process of cell division is critical to the growth and development of an organism. As a fertilized egg develops into a mature organism, tissues undergo cellular renewal or commit to terminal differentiation and leave the cell cycle. Tight regulation of events controlling the cell cycle ensures the integrity of the genetic information and prevents aberrant or unscheduled cell division [1]. Identifying the molecular nature of the mechanisms that control the cell cycle is crucial, since dysregulation of this process can lead to various diseases, including cancer, autoimmunity or degenerative disorders. However, we are still far from a full understanding of the mechanisms operating in the pathways that control the cell division cycle. This Special Issue is focused on some of the key players contributing to the regulation of cellular proliferation and the cell cycle, and how this regulation is altered in stressful or pathological conditions, such as cancer.

As the cell progresses through the interphase of the cell cycle, all the cellular organelles in addition to DNA must be duplicated before the correct partitioning into daughter cells. This process is tightly regulated and probably involves many transcriptional and posttranscriptional networks. However, how cellular organelles are regulated to duplicate their content and size before cell division is not well understood. The group led by Dr. Zubiaga presents evidence that genes encoding proteins residing in the Golgi complex are cell cycle-regulated [2]. Transcription factors of the E2F (E2 Promoter Binding Factor) and the CREB/ATF (cAMP Response Element-Binding protein/Activating Transcription Factor) families were found to regulate transcription of the Golgi-specific *GOLPH3* (Golgi Phosphoprotein-3) gene during G0 and G1 phases of the cell cycle through distinct elements present in its promoter. Interestingly, GOLPH3 levels must be tightly regulated for efficient cell cycle progression, suggesting that a coordinated action of nuclear and organelle components is necessary for a timely cell cycle.

A key player in cell cycle regulation is c-MYC (Avian Myelocytomatosis Viral Oncogene Homolog), a transcription factor commonly overexpressed in human tumors. c-MYC overexpression is thought to disrupt checkpoint control and to promote aberrant cell cycle progression. The specific role of c-MYC in the functional inactivation of cell cycle inhibitors is reviewed by Dr. León and coworkers [3]. Current research is unraveling a variety of mechanisms by which c-MYC modulates the levels of the Cyclin Dependent Kinase Inhibitors 1A (p21^CIP1^), 2A (p14^ARF^), 1B (p27^KIP1^) and 2B (p15^INK4B^), to promote cell cycle entry and progression. Some of these c-MYC -mediated mechanisms operate directly at the transcriptional level through distinct promoter elements, such as the repression of the genes encoding p15^INK4B^ and p21^CIP1^, or the activation of the gene encoding p14^ARF^. Other mechanisms are indirect, through the regulation of miRNA molecules targeting p21^CIP1^ or p27^KIP1^ mRNAs, or mediated by other transcription factors that directly regulate p14^ARF^ expression. Clearly, overexpression of c-MYC, a common alteration in cancer, impacts the gene regulatory network of cell cycle inhibitors to promote cellular proliferation.

In contrast to the well-established oncogenic role of c-MYC, certain regulators of the cell cycle, such as the mitotic regulator Polo Like Kinase 1 (PLK1), appear to play both oncogenic and tumor suppressor roles in a context-dependent manner. A review article by Dr. de Cárcer analyzes in detail the functional complexity of PLK1 [4]. A common consequence of both overexpressing and silencing PLK1 seems to be the induction of aneuploidy and chromosomal instability, particularly when PLK1 is overexpressed in combination with other oncogenes. Several PLK1-inhibitory drugs are being tested in clinical trials. However, given the tumor suppressive activity of PLK1, the author suggests that PLK1-targeting strategies need to be re-evaluated to define what tumors will truly benefit from these approaches.

The cell cycle is endowed with a control system that ensures proper cell division. Stressful or damaging conditions inflicted to cells result in the activation of checkpoints along the cell cycle, which induce cell cycle arrest until the defects are repaired [5]. Dr. Calabrò and collaborators have now analyzed the cellular responses to damage induced by oxidative stress [6]. They have gathered evidence that oxidative stress causes enhanced secretion of YB-1, a multifunctional protein known to accumulate not only in cytoplasmic stress granules, but also in the nucleus, where it participates in DNA repair. In its secreted form, however, YB-1 was found to promote G2/M cell cycle arrest of neighboring cells, a process that was associated with an induction of p21^CIP1^ expression. The authors conclude that oxidative stress not only impacts the damaged cell, but it can be propagated to neighboring cells through paracrine mechanisms involving secreted proteins such as YB-1.

Oxidative stress can be triggered by arsenic trioxide, a first-line chemotherapeutic drug used in oncological practice [7]. Treatment of breast cancer MCF-7 cells with this compound leads to G2/M cell cycle arrest and apoptosis, associated with deactivation of MAPK and PI3K survival pathways. Dr. Mbita and collaborators report in this issue that these processes are accompanied by a complex regulation of survivin gene expression at the level of mRNA splicing [8]. Survivin is an antiapoptotic protein involved in cancer development [9]. Several survivin isoforms can be generated though alternative splicing. Interestingly, one of these isoforms, survivin 2B, is upregulated only when oxidative stress induces G2/M arrest, but not apoptosis, suggesting that this survivin variant may be specifically involved in cell cycle arrest.

Inflicting damage to the tumor cells constitutes the main objective of anticancer chemotherapies. Upon extensive damage, tumor cells respond by inducing apoptosis. However, most chemotherapeutic agents are not only toxic to tumor cells, but also to normal cells, leading to severe side effects. Many laboratories are focusing their efforts on screening probiotics, searching for new compounds with anticancer activity but less overall toxicity. According to the work of Dr. Chung and collaborators in this issue [10]—p8 protein—a small molecule derived from *Lactobacillus,* appears to have antiproliferative activity on colorectal cancer cells, particularly when p8 is expressed ectopically in these cells. p8 is able to induce p21^CIP1^ very efficiently and to reduce cycB1/CDK1 protein levels, thereby arresting the cell cycle in G2/M. The next step for this potential candidate for anticancer gene therapy will be to design effective vectors for its delivery into tumor cells.

The number of proteins known to play a role in cell cycle regulation continues to rise. Dr. Zhao and coworkers report the identification of Ankyrin Repeat Domain 45 (Ankrd45) as a novel cell cycle regulator [11]. Thorough studies in zebrafish embryos and human cell cultures show that Ankrd45 is required for cell proliferation, since its depletion leads to apoptosis. Ankrd45 is mainly localized to the cleavage furrow and the midbody during mitosis, suggesting that it is required for cell division in early and late mitosis. Future research should decipher the mechanism underlying Ankrd45-regulated mitosis and its potential role in cell cycle control.
